# Two Electrical Engineers, One Problem, and Evolution Produced the Same Solution: A Historical Note

**DOI:** 10.16910/jemr.14.1.2

**Published:** 2021-02-15

**Authors:** Louis F. Dell’Osso

**Affiliations:** Daroff-Dell’Osso Ocular Motility Laboratory¹, Louis Stokes Cleveland Department of Veterans Affairs Medical Center and CASE Medical School; and the Department of Neurology², Case Western Reserve University and University Hospitals Cleveland Medical Center; Cleveland, OH. ¹ Director Emeritus; ² Professor Emeritus, USA

**Keywords:** step response, pulse-step, saccades, low-order plants

## Abstract

This note adds historical context into solving the problem of improving the speed of the step response of a low-order plant in two different types of control systems, a chemical mixing system and the human saccadic system. Two electrical engineers studied the above problem: one to understand and model how nature and evolution solved it and the other to design a control system to solve it in a man-made commercial system. David A. Robinson discovered that fast and accurate saccades were produced by a pulse-step of neural innervation applied to the extraocular plant. Leonidas M. Mantgiaris invented a method to achieve rapid and accurate chemical mixing by applying a large stimulus for a short period of time and then replacing it with the desired steady-state value (i.e., a “pulse-step” input). Thus, two humans used their brains to: 1) determine how the human brain produced human saccades; and 2) invent a control-system method to produce fast and accurate chemical mixing. That the second person came up with the same method by which his own brain was making saccades may shed light on the question of whether the human brain can fully understand itself.

## Introduction

At approximately the same time (circa early 1960’s) two classically trained electrical engineers, unknown to each other, were studying similar problems. One, well known to readers of this Journal, was David A. Robinson who was studying how the saccadic subsystem of the ocular motor system (OMS) achieved simultaneously rapid and accurate changes in eye position. The other, unknown to most/all of the readers of this Journal, was Leonidas M. Mantgiaris, who was trying to design a commercial control system that could rapidly and accurately control a chemical mixing plant. The key findings of each will be summarized and compared in this historical note.

## Methods

Robinson combined the control-systems approach with the tools of neurophysiology to identify and model the neurological control signals responsible for the generation of saccadic eye movements. Part of the latter led him to postulate the existence of a neural integrator that transformed the input pulse into a steady-state eye-position signal.

Mantgiaris used the principles of control systems to design a dual-mode controller that used a high input signal for rapid response that was then switched to an integrated steady-state input to maintain an accurate output.

## Results

**Figure 1.**
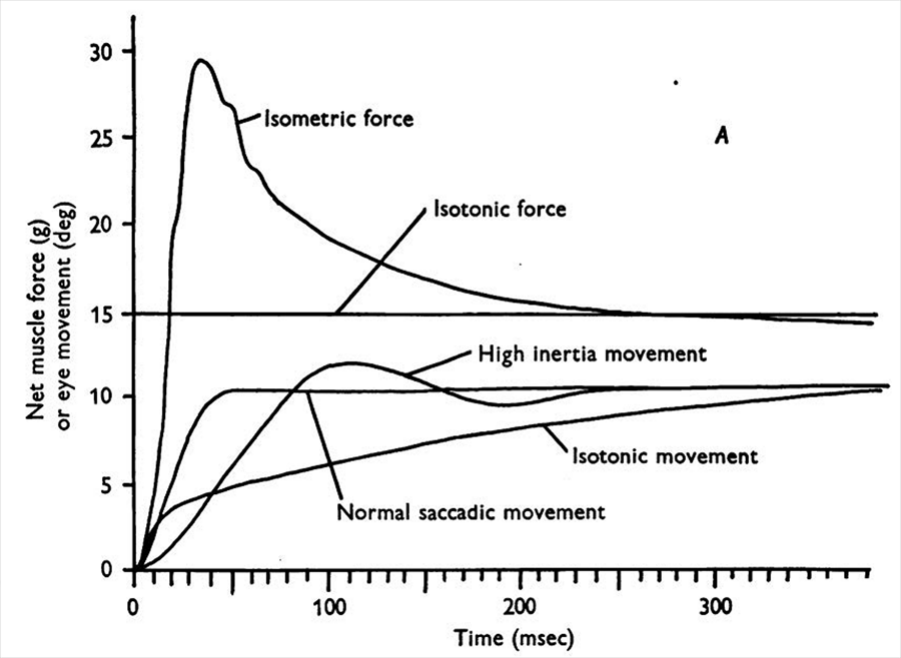
The time courses of isometric, isotonic, high inertia, and normal experiments for a 10° saccade super-imposed. Part B of this Figure (not shown) shows the innervation used. From (1).

In his studies of saccadic eye movements, Robinson began at the plant (the extra-ocular muscle, EOM). He studied the static and dynamic tensions during a saccade and related them to the length-tension curves of the muscle. He noted that despite the size of a saccade, there was a short period of high tension followed by a steady-state tension to maintain eye position (1, 2, 3, 4, 5). It was the duration of this high pulse of tension that determined the saccadic size. Figure 1 illustrates the relationship between isometric, isotonic, and high inertia tensions in EOM during a saccade. The driving signal for a saccade was a “pulse-step” of neural innervation. The sources of the pulse were the burst neurons in the brain stem but the source of the steady-state position signal was unknown. Based on his modeling attempts, Robinson hypothesized that somewhere in the brain was a group of neurons that accomplished the mathematical function of time integration, i.e., a “neural integrator (NI).” This pulse generator + neural integrator combination was adopted by most OMS modelers (including this author) despite the absence of neurophysiological evidence for a neural integrator (6).

**Figure 2.**
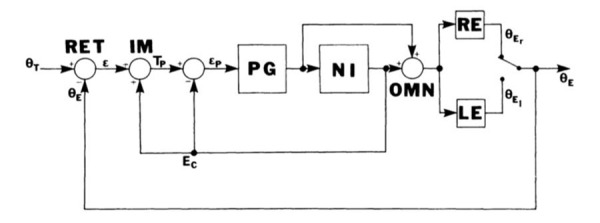
Typical incorporation of Robinson’s pulse generator (PG) and neural integrator (NI) into a model of saccadic eye movements. . MLF, medial longitudinal fasciculus. From (9).

Figure 2 shows Robinson’s saccadic model including the pulse generator (PG) and NI. Although he used a single-pole plant, later models used a 2-pole plant. It would be about two decades until that evidence was produced for neural integration occurring in the nucleus prepositus hypoglossi and Robinson’s intuitive hypothesis supported (7, 8).

**Figure 3.**
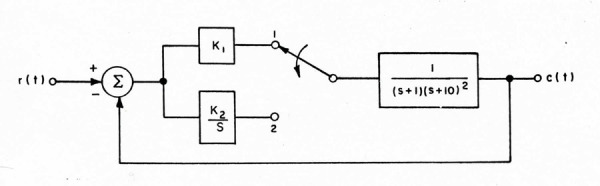
The dual-mode model used by Mantgiaris to achieve both rapid and accurate step responses by a second-order plant. From (11).

In 1962, Mantgiaris began his studies into designing “a simple compensation scheme” so that “the process output closely approximate the process input when the latter is a step and that there be no steady state error.” The reference process was that of a chemical concentration control. His solution was to combine a short pure gain to achieve a fast response followed by integral compensation to assure no steady-state error (10, 11). A switching arrangement was evolved to combine the desirable characteristics of both types of input. Figure 3 is the model shown with a second-order plant.

**Figure 4.**
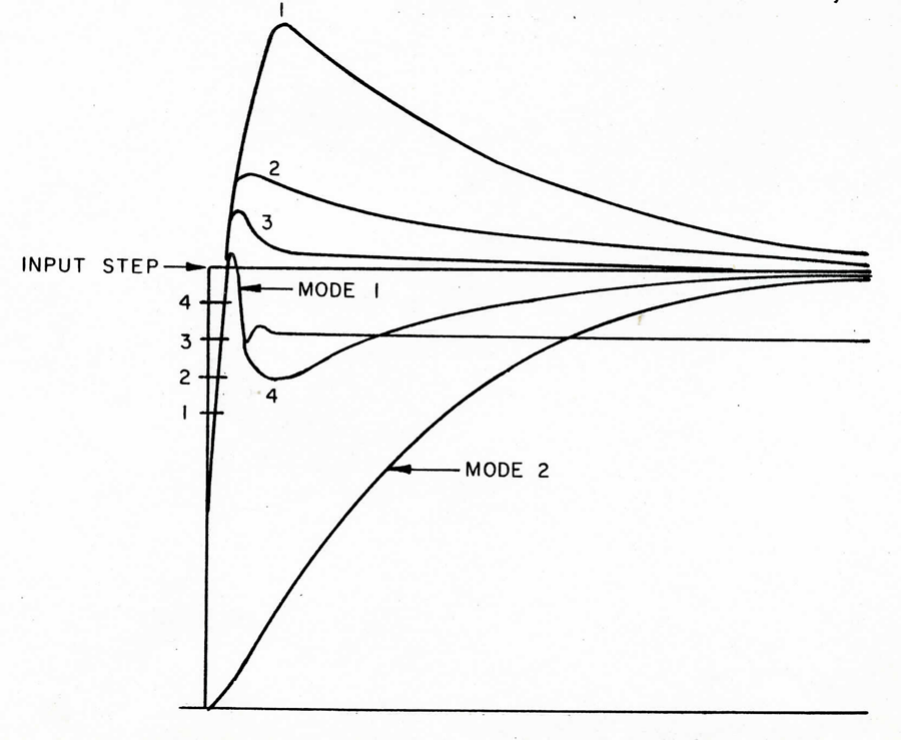
Illustrations of the effects of different switching times on the step response of the dual-mode system. From (11).

Responses from this model for different switching points (i.e., “pulse” widths) are shown in Figure 4. This mode switching between a pulse and a step results in a similar drive to the plant as Robinson’s summing junction with a step feed forward. Amazingly, Mantgiaris also demonstrated how his design allowed accurate following of a constant-velocity (“ramp”) input, thereby anticipating the “step-ramp” method used by OMS modelers of the human smooth pursuit system.

## Discussion

These two electrical engineers were studying essentially the same problem, albeit in far different settings. That is, how can a control system produce a response to a step input applied to a low-order plant that was both rapid and accurate, with neither overshoot nor steady-state error? Each was armed with a thorough knowledge of control-systems analysis and design. Robinson applied that approach to the understanding and modeling of an existing biological control system (saccadic) to identify the neurological signals responsible for generating very fast and accurate saccadic eye movements. Mantgiaris, on the other hand, set out to design and synthesize a commercially useful control system that could provide both a rapid and accurate response to the mixing of two chemicals. Both succeeded and both arrived at the same mechanism; apply a short-lived, large initial drive signal and then replace it with a steady-state input to maintain the desired output (i.e., a pulse-step input). Control-systems analysis has general utility across different physical systems.

I find it fascinating that Robinson postulated the existence of a neural integrator in the brain to generate the steady-state position signal from the pulse. He did so based solely on his model since he had no neurophysiological data to support the existence of such a neural network. He had documented only the burster neurons to produce the pulse and the pulse-step signal at the extraocular muscles. Equally fascinating, albeit unknown to Mantgiaris, the control system that he designed was based on the same principles that were already present in his own brain, enabling him to make fast and accurate saccadic eye movements. Thus, we had one investigator (Robinson), trying to discover how the brain accomplishes a simple ocular motor task, and the other (Mantgiaris) solving a man-made problem, both postulating/using the same ocular motor mechanism that nature and evolution arrived at hundreds of thousands of years ago. Because of my chosen area of research and my close friendship with Mantgiaris, I was fortunate to be able to read both of their research papers when they were written and to discuss their findings with each.

Is it possible that in attempting to solve their research problems, they somehow tapped into how their own brains handled the same problem thousands of times per day? I (probably along with many other “brain” researchers) have always wondered if we scientists, using only our own brains, could *ever* understand the complexities of the human brain. That is, can the brain understand its own complexity? Our models are necessarily far less complex than the parts of the brain they model and similar models may represent different physical systems. I recently opened a fortune cookie that addressed the question in the opposite manner; it read, “If the brain were so simple we could understand it, we would be so simple we couldn’t.” I cannot answer this question but the work of these two insightful investigators, working independently on unrelated control systems, may have shed some light on both the predicament posed by our attempts to understand how our own brains work and a possible source of perspicacity that could provide a pathway to solutions.

## Dedications

### David A. Robinson (1924 – 2016)

I first met David sometime in the late 1960’s or early 1970’s when our respective interests in ocular motor control caused our paths to cross. In the ensuing decades we met many times at scientific congresses and visits to each other’s labs and even traveled together in the US and abroad; we were both colleagues and friends. Having been trained as electrical engineers, we spoke the same language and approached our OMS studies from the same control-systems perspective. This note is dedicated to David and his seminal work in basic ocular motor control.

### Leonidas Miltiadis Mantgiaris (1940 – 1965)

I first met “Lenny” sometime in the 1950’s since we lived only three blocks from each other in Brooklyn. We quickly became close (“best”) friends through grade school, high school (he, Peter Stuyvesant and I, Brooklyn Tech), college (both, Brooklyn Polytechnic Institute), and graduate schools (both, Brooklyn Polytechnic Institute then I, University of Wyoming). We both arranged to become engaged on the same evening at separate dinners with our fiancés and then to meet afterwards so we could enjoy watching the two women rush towards each other with their left hands extended; we were each other’s “best man” at our respective weddings. Needless to say, his untimely death at the age of 25 was devastating to his wife and family, to me, and to all his close friends. He was named for a great Spartan warrior king and an Athenian general, both of whose battlefield valor’s against the Persians saved the Greek people; he was just beginning to live up to his namesakes. This note is dedicated to Lenny and to the unfulfilled promise of significant contributions that I am confident he would have made.

### Ethics and Conflict of Interest

The author declares that the contents of the article are in agreement with the ethics described in http://biblio.unibe.ch/portale/elibrary/BOP/jemr/ethics.html and that there is no conflict of interest regarding the publication of this paper.

### Acknowledgements

This work was supported in part by the Office of Research and Development, Medical Research Service, Department of Veterans Affairs.
